# Brain Tumor Segmentation Based on Hybrid Clustering and Morphological Operations

**DOI:** 10.1155/2019/7305832

**Published:** 2019-04-09

**Authors:** Chong Zhang, Xuanjing Shen, Hang Cheng, Qingji Qian

**Affiliations:** ^1^College of Software, Jilin University, Changchun, China; ^2^Key Laboratory of Symbolic Computation and Knowledge Engineering of Ministry of Education, Jilin University, Changchun, China; ^3^College of Computer Science and Technology, Jilin University, Changchun, China; ^4^Department of Pediatrics, The First Hospital, Jilin University, Changchun, China; ^5^College of Physics, Jilin University, Changchun, China

## Abstract

Inference of tumor and edema areas from brain magnetic resonance imaging (MRI) data remains challenging owing to the complex structure of brain tumors, blurred boundaries, and external factors such as noise. To alleviate noise sensitivity and improve the stability of segmentation, an effective hybrid clustering algorithm combined with morphological operations is proposed for segmenting brain tumors in this paper. The main contributions of the paper are as follows: firstly, adaptive Wiener filtering is utilized for denoising, and morphological operations are used for removing nonbrain tissue, effectively reducing the method's sensitivity to noise. Secondly, K-means++ clustering is combined with the Gaussian kernel-based fuzzy C-means algorithm to segment images. This clustering not only improves the algorithm's stability, but also reduces the sensitivity of clustering parameters. Finally, the extracted tumor images are postprocessed using morphological operations and median filtering to obtain accurate representations of brain tumors. In addition, the proposed algorithm was compared with other current segmentation algorithms. The results show that the proposed algorithm performs better in terms of accuracy, sensitivity, specificity, and recall.

## 1. Introduction

**Figure 1:**
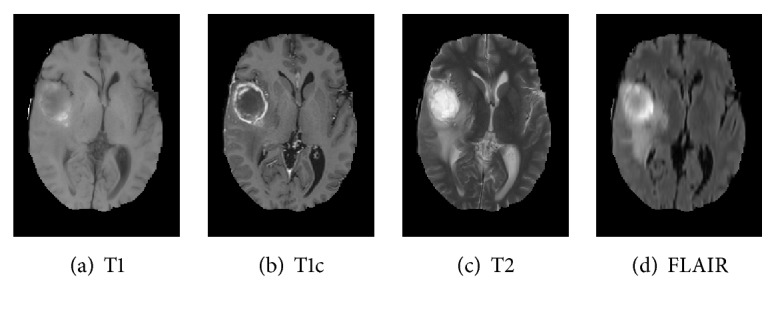
Brain MRI images containing tumors in four different modalities.

Brain tumor is one of the most serious diseases, which often have lethal outcomes. At present, more and more attention has been paid to the study of brain tumor image. Nowadays, MRI is especially useful for brain imaging [1], which can be performed without injecting radioisotopes. MRI is based on multiparameter imaging, which can form different images by adjusting different parameters and contains a large amount of information.  Figure 1 exemplifies brain MRI with tumors, and the images were obtained in four different modalities: T1, T1c, T2, and FLAIR. The FLAIR modalities are usually used for finding the extensions of tumors and edemas. Here, we use segmentation of FLAIR images in BRATS 2012 [2].

As shown in Figure 1, MRI images usually have low contrast, and it is difficult to diagnose lesion areas owing to noise accurately. Therefore, accurate tumor segmentation is essential. Nowadays, many image segmentation techniques have been widely applied to segmentation of medical images. Examples include the threshold segmentation algorithm [3], edge-based segmentation algorithms [4], and neural network-based segmentation [5]. However, there is no efficient and versatile method of brain tumors based on imaging.

The threshold-based segmentation algorithm determines the segmentation threshold based on certain pixel features. The pixels' feature values are compared with the segmentation threshold to determine which parts of the image to categorize the pixels. This method is simple to implement and execute.

Since the characteristics of the boundary pixels are discontinuous, the pixel features on both sides of the boundary will have relatively obvious differences. Therefore, the basic idea of the edge-based segmentation algorithm is to find the boundaries using some method and to specify the directions of the boundary first. Then, the pixels on one side of the boundary are divided into one subimage, while the pixels on the other side are considered to belong to another subimage. Although this algorithm is fast, it is sensitive to noise and usually obtains incomplete information.

In recent years, image segmentation using neural networks has become increasingly popular. The basic idea in this approach is to train a neural network on a training set and then modify the architecture and weights of connections between the network's nodes. New image data are segmented using a trained neural network. Convolutional neural networks (CNNs) have been particularly popular among different neural network methods [5]. Yet, one of the most difficult issues related to neural networks is constructing the network. Neural networks are computationally intensive and time-consuming, which hinders implementation.

Clustering algorithms are commonly used for segmentation of medical images. Commonly used clustering algorithms include fuzzy C-means clustering (FCM), K-means clustering, and expectation maximization (EM) [6–8]. The K-means algorithm is a hard clustering algorithm, which iteratively calculates the gray scale means of different clusters, computes the distances from the image's pixels to the clusters' centroids, and assigns the image's pixels to classes that correspond to the nearest centroid. Fuzzy C-means clustering utilizes the fuzzy set theory, which allows soft segmentation. The EM algorithm assumes that data can be described as a mixture of probability distributions. Then, the algorithm iteratively calculates the posterior probability and estimates the mean, covariance and mixture coefficients using the maximal likelihood estimation approach and clustering criteria [9]. However, this clustering algorithm is sensitive to noise.

In order to improve the instability clustering and to alleviate its sensitivity to noise, an effective clustering segmentation algorithm is proposed in this paper. The main contributions of this paper are as follows:

- A hybrid clustering algorithm based on K-means++ and Gaussian kernel-based fuzzy C-means (K^++^GKFCM) is proposed.
- K-means++ algorithm is utilized to initialize the clustering center, which greatly improves the stability of the algorithm.
- Gaussian kernel-based fuzzy C-means is proposed, which improves the sensitivity to noise.
- The proposed method is combined with morphological operations for preprocessing and postprocessing, which further improves the accuracy of segmentation.

As a result, the accuracy of image segmentation is significantly improved.

The remainder of the paper is organized as follows: Section 2 depicts the related work of the paper.  Section 3 details the methods used in this article.  Section 4 presents the experimental results and assessments. Finally, conclusions and outstanding issues are listed in Section 5.

## 2. Related Work

Segmentation of medical images is a very popular research topic, and many methods have been developed. Clustering algorithms for image segmentation are very popular among scholars, and many of these algorithms have been employed for image segmentation. Dhanalakshmi and Kanimozhi [10] proposed an algorithm for automatic segmentation of brain tumor images based on K-means clustering. During preprocessing, a median filter is used to remove artifacts and sharpen the image's edges. Seed points are randomly selected for K-means in this method. A binary mask is applied for identification of high-contrast categories. However, K-means clustering is more affected by abnormal points and is more sensitive to initialization.

Kalaiselvi and Somasundaram [11] applied fuzzy C-means (FCM) to segmentation of brain tissue images, which is computationally more efficient owing to the initialization of seed points using the image histogram information. Yet, this method still does not address the sensitivity to noise and intensity inhomogeneity (IIH). Noreen et al. [12] introduced a hybrid MR segmentation method based on the discrete wavelet transform (DWT) and FCM for removal of inhomogeneity. This method applies the DWT to the input MR image, to obtain four subbands; then, the inverse discrete wavelet transform (IDWT) is applied to obtain a high-pass image. Finally, FCM clustering is performed to segment the image. Although this method addresses the sensitivity problem of intensity nonuniformity, it does not consider the uncertainty of the data space information. Christe et al. [13] combined K-means with fuzzy C-means. They defined the number of clusters, ambiguity, distance, and stopping criteria. Their method can handle overlapping intensities, but it cannot clearly define tissue boundaries. Wilson and Dhas [14] used K-means and FCM to detect iron in brain SWI, and compared the two algorithms. The experimental results showed that the FCM algorithm is better at detecting iron-containing regions, compared with K-means. Abdel-Maksoud et al. [15] reconsidered the advantages and disadvantages of K-means clustering and FCM clustering. They also proved that the K-means algorithm can detect brain tumors faster than the FCM algorithm, while the FCM algorithm can detect tumors that are not detected by K-means. They proposed to combine K-means clustering with FCM for segmentation. Their experimental results showed that the combination of the two algorithms is more advantageous than the individual algorithms. The disadvantage of this approach is that the two algorithms select their seed points in a random manner, which can easily result in overfitting.

Chuang et al. [16] proposed to add spatial information to the FCM algorithm and update the membership function twice, which significantly improved the effect of FCM clustering. On this basis, Adhikari and Sing [17] introduced the conditional space fuzzy C-means (csFCM) clustering algorithm. The underlying idea is to apply an adjustment effect to the auxiliary variables corresponding to each pixel, which effectively reduces the algorithm's sensitivity to noise and intensity nonuniformity with respect to MRI data. Bai and Chen [18] proposed an improved FCM segmentation algorithm based on the spatial information for infrared ship segmentation (sFCM), which introduced improvement from the viewpoint of the following two aspects: (1) addition of nonlocal spatial information based on ship targets (2); refining of the local space constraints through the Markov random field using the spatial shape information of the ship's target contour. Ghosh and Mali et al. [19] put forward a new FCM clustering application, which uses the firefly algorithm and a chaotic map to initialize the firefly population and adjusts the absorption coefficient to improve the mobility of global search. The algorithm is called C-FAFCM. Al-Dmour and Al-Ani [20] proposed a fully automatic algorithm for brain tissue segmentation, based on the clustering fusion methodology. They combined three clustering techniques (K-means, FCM, and self-organizing map (SOM)) with neural network models for training and testing. Classification was performed using a voting strategy, which significantly improved the algorithm's segmentation performance. Still, the stability of the algorithm remained unresolved.

Although the current medical image segmentation algorithm reduces the sensitivity of noise to some extent, the stability of segmentation is still a huge challenge. For the purpose of alleviating the sensitivity of the clustering algorithm to noise and for improving the stability of the clustering algorithm, here we propose to the K^++^GKFCM algorithm, benefitting from the advantages of the two clustering algorithms. In addition, morphological operations are applied for preprocessing and postprocessing, to further improve the accuracy of segmentation. Finally, the proposed method is compared with the K-means algorithm, the FCM algorithm, and the improved clustering algorithm in recent years. The results of this comparison show that the proposed algorithm performs better.

## 3. Proposed Method

**Figure 2:**
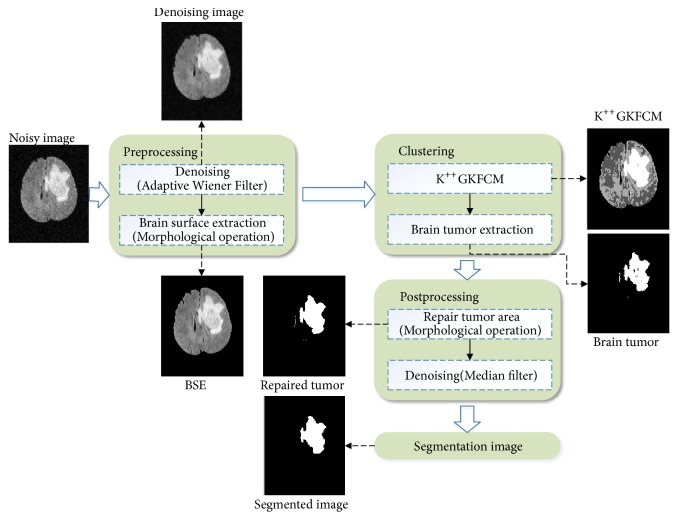
The framework of the algorithm proposed in this paper.

As shown in Figure 2, the segmentation algorithm proposed in this paper is mainly divided into three parts.

**Step 1 (preprocessing and completing the brain surface extraction (BSE)).**

The original noisy brain MR image is denoised using adaptive Wiener filtering, and the part corresponding to the skull is removed by morphological operations, to obtain a denoised image of brain parenchyma.

**Step 2 (clustering and extraction of the tumor image).**

K^++^GKFCM are used for cluster segmentation. The tumor region is extracted according to a threshold.

**Step 3 (postprocessing).**

Morphological operations and median filtering are applied as postprocessing to obtain the final segmentation results.

### 3.1. Preprocessing and Morphological Operations

**Figure 3:**
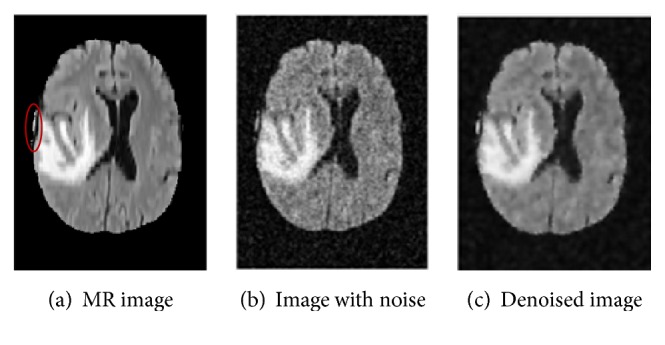
Image denoising, accomplished by adding noise and using adaptive Wiener filtering.

Medical images are often noisy, which greatly affects segmentation of lesions and diagnosis of patients' conditions. In this paper, adaptive Wiener filtering is used for denoising MRI brain images; this filtering allows to effectively eliminate Gaussian noise, while protecting the texture of the original image.  Figure 3 shows an image with Gaussian noise (noise variance, 0.02) and the corresponding denoised image obtained using adaptive Wiener filtering.

Furthermore, MR brain images often contain images of nonbrain tissues such as the skull and outer membrane, as shown in red in Figure 3. To reduce computational complexity and improve segmentation, morphological operations are utilized for removal of nonbrain tissue. Morphological operations are utilized for identifying the boundaries and skeletons of objects in an image [21]. The most common morphological operations are expansion and corrosion. Expansion enlarges the image's edges, filling the edges of the target or its internal depression. Corrosion erodes the image's boundaries [22]; the goal is to erode the sawtooth of the target's edges. The opening operation is an extension of the expansion and corrosion operations, where etching is performed first and then the same structural elements are used for expansion [23]; this operation is denoted as *X*∘*Y* and is defined by

(1) $$\begin{array}{c} X\circ Y=\left(\;X⊝Y\;\right)⊕Y \end{array}$$

where* X* is the image of the brain,* Y* is the structural element, “∘” means the corrosion operation, and “⊕” means the expansion operation.

The morphological opening operation is applied to remove the images of nonbrain tissue from the MR brain image, and the hole-filling technique is used for repair, to obtain a complete brain parenchymal region. The purpose of this step is to reduce the complexity of the algorithm, and the accuracy of the proposed clustering algorithm is also improved to some extent.

**Figure 4:**
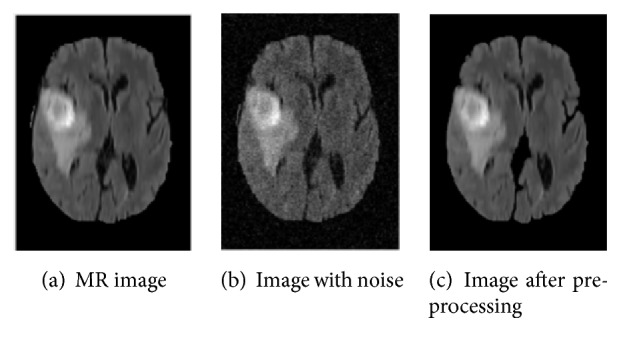
Example of adding Gaussian noise (variance, 0.02) to the MR image for denoising and the resulting image obtained after preprocessing.

For example, consider a random MR image, to which Gaussian noise (variance, 0.02) was added, as shown in Figure 4(b). Using the above-described preprocessing steps, the noise and nonbrain structures are effectively removed, while at the same time the texture features of the MR image are preserved. The result of this preprocessing is shown in Figure 4(c).

### 3.2. Cluster Segmentation and Postprocessing

The proposed K^++^GKFCM clustering algorithm first uses K-means++ for deterministic initialization of cluster centroids to avoid overfitting and then uses the Gaussian kernel-based fuzzy C-means algorithm to perform clustering, which further improves the classification ability.

The classical K-means algorithm accepts the set of samples (data), the number of clusters* k* into which to partition the data, and the maximal number of iterations* N*; the algorithm outputs data classification into clusters [24]. The K-means algorithm is simple and easy to operate, but it also has certain drawbacks. Firstly, the number of cluster centroids* k* in the K-means algorithm needs to be specified in advance, which significantly limits treating unknown data (with unknown number of clusters). Secondly, before clustering using the K-means algorithm,* k* cluster centroids need to be initialized, and, typically, numbers ranging from minimal to maximal values of data are selected randomly as data centroids. However, the choice of cluster centroids may significantly affect the clustering classification of the K-means algorithm.

In classical clustering algorithms, whether K-means or FCM, cluster centroids are uncertain. There are three methods to initialize cluster centroids, (1) K-means; (2) K-means++; (3) clustering with the hierarchical clustering or Canopy algorithm, and then select a point from each cluster, which may be the cluster centroid or the closest point to the cluster centroid. The traditional K-mean algorithm randomly selects* k* clustering centers, which has poor clustering effect. The latter two methods have similar effects, but the complexity of K-means++ is lower and the method is easy to implement. Thus, K-means++ is adopted to initialize the cluster centroid in this paper.

K-means++ is based on K-means, which can initialize the centroids deterministically. The basic principle of the K-means++ algorithm for initialization of cluster centroids is to maximize the distance between the initial cluster centroids. This method allows deterministically initializing cluster centroids, overcoming the shortcomings of the K-means algorithm associated with its initialization instability [25, 26]. The initialization process of the K-means++ algorithm is as follows:

(1) Randomly select a sample point from the data set as the first initialized cluster centroid.

(2) Select the remaining cluster centroids:

(a) Calculate the distance between each sample point in the sample and the cluster centroid that has been initialized, and then select the shortest distance among them, denoted as *d*_*i*_.

(b) Select the sample with the largest distance by probability as the new cluster centroid.

(c) Repeat the above process until k cluster centroids are determined.

(3) For the *k* initial cluster centroids, the final cluster centroids are calculated using the K-means algorithm.

In addition, we introduce the Gaussian kernel method based on the original FCM algorithm [27, 28]. The traditional FCM algorithm dismisses the hard clustering paradigm by introducing the concept of a fuzzy set. The so-called fuzzy set can be defined as follows: Let *M* be the mapping of a set* X* to [0,1], with the mathematical operation expressed as *M* : *X* → [0,1], *x* → *M*(*x*), where *M*(*x*) is the membership function of the fuzzy set *X*. Then,* X* is said to be a fuzzy set on *M*. The FCM algorithm divides the *X* pixels in the image *T* into *c* fuzzy clusters, finding the cluster centroid of each fuzzy cluster and obtaining the objective function [29, 30] by iteration. The objective function can be expressed as

(2) $$\begin{array}{c} \min J_{FCM}=\overset{c}{\underset{i=1}{\sum }}\;\overset{N}{\underset{j=1}{\sum }}u_{ij}^{n}{\left\|\;x_{j}-v_{i}\;\right\|}^{2} \end{array}$$

where *x*_*j*_ represents the* j*-th pixel, v_*i*_ represents the* i*-th cluster centroid, *u*_*ij*_ represents the membership degree of *x*_*j*_ in the* i*-th fuzzy cluster, the constraint is given by (3), and* n* is the fuzzy index, which controls the algorithm's flexibility. The value of* n* affects clustering. The cluster centroid v_*i*_ and the corresponding membership degree *u*_*ij*_ can computed from (4) and (5)

(3) ∑i=1Cuij=1,0≤uij≤1

(4) vi=∑j=1Nuijnxj∑j=1Nuijn

(5) uij=∑k=1Cxj−vixj−vk2

Based on the traditional FCM algorithm, a fuzzy clustering algorithm based on a Gaussian kernel is introduced to efficiently reduce the sensitivity of the algorithm's scaling parameter. The objective function can be expressed as

(6) $$\begin{array}{c} J_{KFCM}=\overset{c}{\underset{i=1}{\sum }}\;\overset{N}{\underset{j=1}{\sum }}u_{ij}^{n}{\left\|\;\varphi \left(\;x_{j}\;\right)-\varphi \left(\;v_{i}\;\right)\;\right\|}^{2} \end{array}$$

where *φ*(*x*) is a nonlinear mapping with constraints as in

(7) φxj−φvi2=φxj−φviTφxj−φvi=φxjTφxj−φviTφxj−φxjTφvi+φviTφvi=Kxj,xj+Kvi,vi−2Kxj,vi

Here, *K*(*x*, *y*) is the inner product of the kernel function. Furthermore, *K*(*x*, *y*) can be expressed as *K*(*x*, *y*) = *φ*(*x*)^*T*^*φ*(*y*), which has the property *K*(*x*, *x*) = 1. Therefore, the target function is given by

(8) $$\begin{array}{c} J_{K^{++}GKFCM}=2\overset{c}{\underset{i=1}{\sum }}\;\overset{N}{\underset{j=1}{\sum }}u_{ij}^{n}\left(\;1-K\left(\;x_{j},v_{i}\;\right)\;\right) \end{array}$$

Since a Gaussian kernel is introduced in this paper, *K*(*x*, *y*) here can be defined as in

(9) $$\begin{array}{c} K\left(\;x,y\;\right)=\exp\left(\;-\frac{{\left\|x-y\right\|}^{2}}{2\sigma^{2}}\;\right)\;\sigma \in R\text{ and}\;\;\sigma \neq 0 \end{array}$$

Here, we need to choose a suitable Gaussian parameter *σ*^2^ to ensure accurate clustering. The pseudocode of the proposed method is shown in Table 1.

Using the clustering algorithm shown in Table 1, four functionally different regions are segmented: (1) gray matter, (2) white matter, (3) cerebrospinal fluid, and (4) tumor and edema areas. We extracted edema and tumor areas using thresholding.

**Figure 5:**
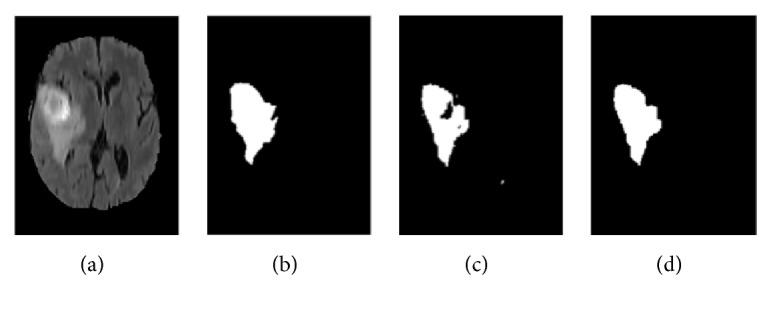
Results of segmentation after postprocessing: (a) MR image, (b) ground truth, (c) tumor area extracted without postprocessing, and (d) tumor area obtained after postprocessing.

Due to various factors, such as noise and intensity nonuniformity, the segmented images obtained using the above clustering algorithm may feature small holes or oversegmentation, as shown in Figure 5(c). To improve the accuracy of segmentation, hole-filling and median filtering are used for postprocessing. After the postprocessing, the small holes in the extracted tumor areas are filled and some missegmented areas are filtered.

The results of the segmentation algorithm after postprocessing are shown in Figure 5(d). Furthermore, Figure 5(a) shows the original MR image and Figure 5(b) shows the ground truth image.

## 4. Experimental Classification Results and Analysis

The proposed method is implemented in Matlab R2016a software, which is run on an Intel Core i5 CPU 2.5 GHz with 8 GB of RAM. The algorithm is tested on the BRATS 2012 open source image library (http://www.slicer.org/pages/Special:SlicerDownloads), which contains brain MR images of different modalities. The work described in this paper is used for segmentation of FLAIR images in BRATS 2012. About 100 pairs of MR images of twenty different patients containing tumors are selected for testing the segmentation algorithm.

### 4.1. Testing the Algorithm's Stability and Robustness to Noise

**Figure 6:**
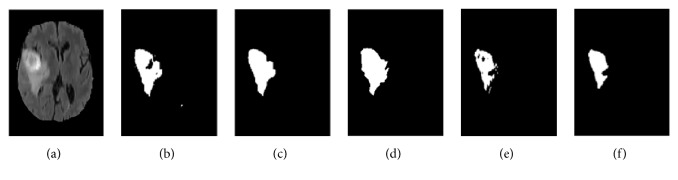
Generation of two unstable results from cluster centroids using K-means: (a) MR image; (b) tumor region extracted from the first result; (c) tumor region after postprocessing extracted from the first result; (d) Ground truth image; (e) tumor region extracted from the second result; (f) tumor region after postprocessing extracted from the second result;

Whether with FCM or K-means clustering, the choice of cluster centroids is uncertain. If K-means is used first for centroid initialization and is then combined with the Gaussian kernel-based FCM clustering algorithm, two different segmentation results are obtained, as shown in Figure 6.

Figure 6 shows the two types of results of the segmentation procedure, where Figure 6(b) shows the first image obtained after postprocessing and Figure 6(c) shows the tumor region extracted using the first clustering result.  Figure 6(e) shows the tumor region extracted using the second clustering result, and Figure 6(f) shows the second result after postprocessing. Figures 6(a) and 6(d) are the original MR image and the ground truth image, respectively.

To improve the stability of the segmentation algorithm, this paper proposes to use K-means++ for deterministic initialization of cluster centroids. Experiments show that the proposed method exhibits very good stability. The specific segmentation results are shown in Table 2.

In addition, MR images are often corrupted by Gaussian noise, which greatly affects medical image segmentation. However, a common disadvantage of conventional clustering algorithms is that they are sensitive to noise. To alleviate this shortcoming, adaptive Wiener filtering and morphological operations are used for preprocessing in this paper. To further verify the robustness of the proposed algorithm to noise, we added Gaussian noise with variances of 0.005, 0.01, and 0.02, to the MR image. Table 2 lists the effect of adding Gaussian noise with the above variances. The segmentation results remain stable across a range of noise variances. It is easy to see that the proposed algorithm is highly robust to noise.

### 4.2. Comparison with Some Recently Proposed Clustering Algorithms

**Figure 7:**
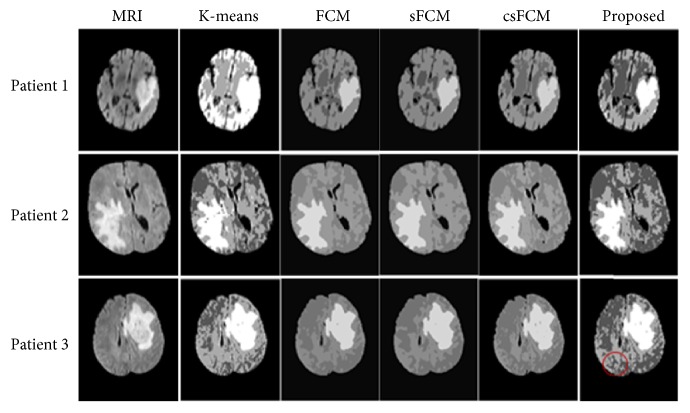
Clustering results of the K-means, FCM, SFCM, and CSFCM algorithms and the proposed clustering algorithm.

Many clustering algorithms have been proposed recently. We compared the proposed method with some commonly used clustering algorithms, to verify the effectiveness of the proposed clustering algorithm. Three brain MR images were randomly selected for analysis.  Figure 7 shows the clustering effect of the proposed algorithm and its comparison to the FCM, K-means, sFCM, and csFCM clustering performances. It is not difficult to see that the algorithm proposed in this paper more accurately treats texture details, compared with the other algorithms. Specifically, the currently proposed clustering algorithm better captures the area marked in red in Patient 3.

To further verify the effectiveness of the proposed algorithm, four evaluation indicators of* Dice*,* Sensitivity*,* Specificity*, and* Recall* were used to evaluate the quality of segmentation. The* Dice* value is the most frequent evaluation index, which indicates the ratio of the area where the two objects intersect to the total area. The* Dice* value for a perfect division is 1.* Sensitivity* quantifies the number of true positives (TPs), pixels that are correctly identified by the algorithm as belonging to the region of interest; higher number of true positives implies higher* Sensitivity*.* Specificity* quantifies the number of false positives (FPs), pixels that in truth do not belong to the region of interest but are classified as belonging to it; higher number of false positives lowers the* Specificity*. The* Recall* is a ratio of TPs to all positives, which is the sum of TPs and false negatives (FNs) [20, 31]. These indicators are calculated as follows:

(10) Dice=TP+TNTP+TN+FP+FN

(11) Sensitivity=TPTP+FP

(12) Specificity=FNTN+FN

(13) Recall=TPTP+FN

where* TP*,* TN*,* FP*, and* FN* are defined as follows:

- *TP* is tumor exists and is detected correctly.
- *TN* is tumor does not exist and is not detected.
- *FP* is tumor does not exist but is detected.
- *FN* is tumor exists but is not detected.

In this paper, brain images of three different patients were taken as examples, for comparison of several clustering algorithms with the proposed algorithm.  Table 3 shows the comparison of the K-means, FCM, sFCM [17], and csFCM [16] algorithms with the proposed algorithm. The proposed algorithm exhibits higher values on the* Dice*,* Sensitivity*, and* Specificity* indicators. However, the* Recall* of the proposed method is slightly lower than those of the FCM, sFCM, and csFCM algorithms.

**Figure 8:**
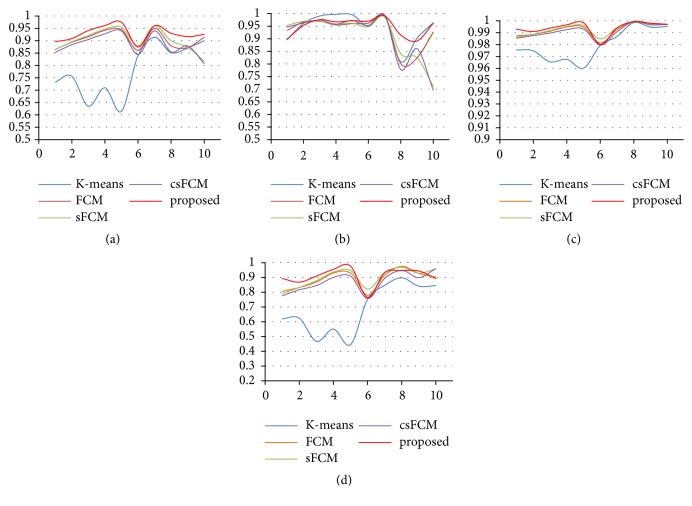
Comparison of four evaluations for the K-means, FCM, sFCM, and csFCM algorithms and the proposed algorithm, for MRI brain images with Gaussian noise with the variance of 0.005: (a) Dice; (b) Sensitivity; (c) Specificity; (d) Recall.

To better quantify the segmentation performance, the segmentation results of 10 pairs of images with Gaussian noise with the variance of 0.005 were randomly selected, for generating curves that correspond to the four evaluations. The results are shown in Figure 8, where the red curve represents the result of the proposed algorithm. Due to the unclear texture of some images, the clustering effect will be reduced. However, except for* Sensitivity* values of some images that are slightly lower than those of some comparison algorithms, the other evaluations are still higher than other comparison algorithms. Compared with other clustering algorithms, the proposed algorithm performs better overall.

To further prove the validity of the algorithm, Table 4 lists the average of the four evaluation indicators for all 100 images.

It is not difficult to see that the proposed algorithm performs better in* Dice*,* Sensitivit*y,* Specificity*, and* Recall*.

## 5. Conclusion

In this paper, a hybrid clustering algorithm combined with morphological operations was proposed for segmentation of brain tumor images. The algorithm first uses morphological operations to remove the outer membrane, which reduces the computational complexity and the number of clustering iterations. In the clustering stage, the K-means++ clustering algorithm is exploited to initialize the clusters' centroids. This method solves the problem of unstable clustering, which arises owing to the uncertainty associated with initialization of cluster centroids. Each cluster only produces a stable clustering result. Furthermore, the proposed method prevents overfitting. Next, the algorithm uses fuzzy C-means clustering based on a Gaussian kernel. The sensitivity to clustering parameters is greatly reduced for the proposed algorithm, and the algorithm's robustness is further improved. Finally, morphological operations and median filtering are applied as postprocessing, which further improves the accuracy of segmentation.

## Figures and Tables

**Table 1:** Pseudocode of the image segmentation procedure.

(1) Input: MR image
(2) Output: Segmented tumor image
(3) Preprocessing: Perform adaptive Wiener filter and
morphological operation.
(4) Set the value of clusters *k*, the degree of fuzziness *m*, the
error *ɛ*, and the value of objective function *J*_*K*^++^*GKFCM*_^(0)^
(5) Initialize the cluster centroid using K-means++:
(6) Choose an initial center *c*_1_ = *x* ∈ *R* at random from image *R*, where *x* represents the pixel of the MR image
(7) Begin
(8) Calculate the probability of each remaining pixel using
*D*(*x*′)^2^/∑_*x*∈*R*_*D*(*x*)^2^, where *D*(*x*) is the distance between the
pixel *x* and the nearest center, *x*' represents the next pixel.
(9) Choose the pixel with the highest probability as the next
initial center
(10) Update the the *i*-th cluster centroid *c*_*i*_
(11) If *k* initial centers are calculated
(12) Then Break
(13) End if
(14) Cluster the obtained images using K^++^GKFCM:
(15) Begin:
(16) Calculate *K(x, y)* using Eq. (9)
(17) Update the membership degree u_*ij*_ using Eq. (5)
(18) Update the the *i*-th cluster centroid v_*i*_ using Eq. (4)
(19) If |*J*_*K*^++^*GKFCM*_^(*i*)^ − *J*_*K*^++^*GKFCM*_^(*i* − 1)^| ≤ *ε*, where *J*_*K*^++^*GKFCM*_^(*i*)^ represents
the function of the *i*-th iteration
(20) Then Break
(21) End if
(22) End

**Table 2:** The effect of adding noise.

Noise variance		MRI	Noisy image	Preprocessing	Clustering	Tumor extraction	Postprocessing
Img1	0.005		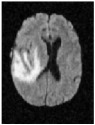	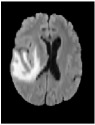	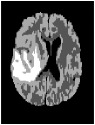	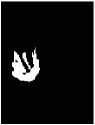	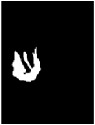
	0.01	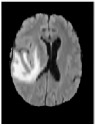	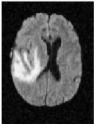	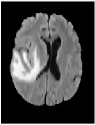	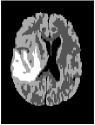	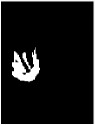	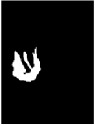
	0.02		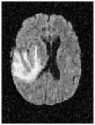	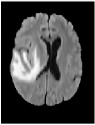	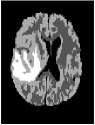	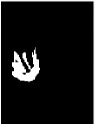	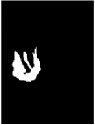

Img2	0.005		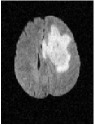	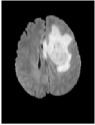	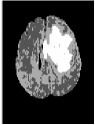	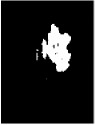	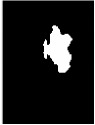
	0.01	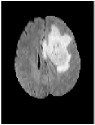	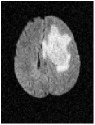	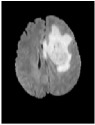	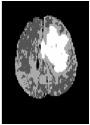	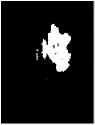	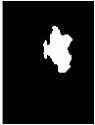
	0.02		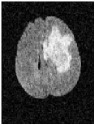	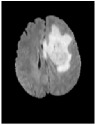	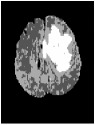	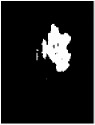	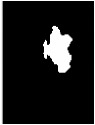

Img3	0.005		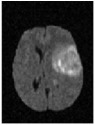	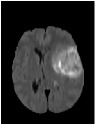	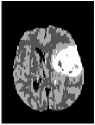	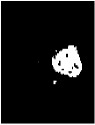	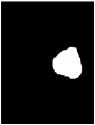
	0.01	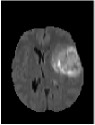	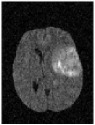	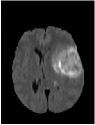	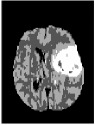	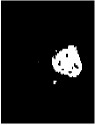	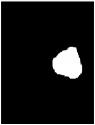
	0.02		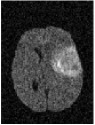	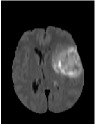	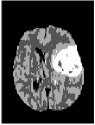	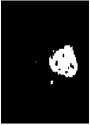	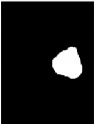

**Table 3:** Comparison of four clustering algorithms and the proposed algorithm.

Clustering methods	Evaluations	Patient 1	Patient 2	Patient 3
K-means	Dice	0.9001	0.9316	0.8298
	Sensitivity	0.9630	0.9064	0.9424
	Specificity	0.9952	0.9984	0.9780
	Recall	0.8449	0.9583	0.7412

FCM	Dice	0.9137	0.9341	0.9004
	Sensitivity	0.9263	0.8905	0.9336
	Specificity	0.9971	0.9994	0.9880
	Recall	0.9015	0.9823	0.8694

sFCM	Dice	0.8169	0.9258	0.9144
	Sensitivity	0.7112	0.8645	0.9290
	Specificity	0.9968	0.9994	0.9878
	Recall	0.9597	0.9965	0.9002

csFCM	Dice	0.8069	0.9179	0.9166
	Sensitivity	0.6960	0.8540	0.9290
	Specificity	0.9971	0.9996	0.9880
	Recall	0.9597	0.9922	0.9045

Proposed	Dice	*0.9261*	*0.9400*	*0.8978*
	Sensitivity	*0.9622*	*0.9184*	*0.9380*
	Specificity	*0.9971*	*0.9996*	*0.9881*
	Recall	*0.8926*	*0.9625*	*0.8608*

**Table 4:** The mean of four clustering algorithms and the proposed algorithm on 100 images.

Clustering methods	Dice	Sensitivity	Specificity	Recall
K-means	0.7988	0.9421	0.9812	0.7159
FCM	0.9061	0.9257	0.9927	0.8931
sFCM	0.9045	0.9097	0.9932	0.9079
csFCM	0.8890	0.9063	0.9916	0.8834
Proposed	*0.9256*	*0.9460*	*0.9941*	*0.9087*

## Data Availability

The data used to support the findings of this study are included within the article
